# Association between physical activity and adolescent mental health in the post COVID-19: The chain mediating effect of self-esteem and social anxiety

**DOI:** 10.1371/journal.pone.0301617

**Published:** 2024-05-17

**Authors:** Wanxuan Feng, Liangyu Zhao, Zhang Ge, Xiuhan Zhao, Tuojian Li, Qiying Zhu

**Affiliations:** School of Physical Education, Shandong University, Lixia District, Jinan City, Shandong Province, China; The Open University of Israel, ISRAEL

## Abstract

**Background:**

In order to gain a deeper understanding of the relationship between physical activity and adolescent mental health in the post COVID-19 pandemic era, self-esteem and social anxiety were used as mediating variables to explore the potential mechanisms by which physical activity affects adolescent mental health.

**Methods:**

The study used the HELP-II Health Promoting Lifestyle Scale, the SPIN Social Phobia Scale, the Self-Esteem Scale, and the 10-item Kessler Psychological Distress Scale to administer questionnaires to 400 Chinese secondary school students, and SPSS 26.0 and PROCESS 3.3 were used to process the data.

**Results:**

The findings showed that (1) physical activity was significantly and positively associated with mental health; (2) self-esteem and social anxiety played a fully mediating role between physical activity and adolescent mental health respectively; (3) self-esteem and social anxiety played a chain mediating role between physical activity and adolescent mental health.

**Conclusion:**

This study reveals the relationship and influencing mechanism between physical activity and adolescent mental health in the post COVID-19 pandemic era. Appropriate interventions for physical activity, self-esteem, and social anxiety may be beneficial to adolescent mental health. The protective role of self-esteem in adolescent mental health should be the focus of future studies, and further investigations into the association between the COVID-19 and adolescent mental health are warranted.

## 1. Introduction

According to a report by the World Health Organization, as of February 4, 2024, there have been over 774 million confirmed cases and over 7 million deaths worldwide [1]. A meta-analysis pointed out that the global prevalence of depression in 2017 was 3.44%, while the prevalence of depression during the COVID-19 was as high as 25%, seven times higher than that in 2017 [2]. A study found that during the COVID-19 pandemic, the level of sports participation among adolescents continued to decline, leading to a continuous increase in mental health problems [3]. In addition, the lack of face-to-face social interaction during the COVID-19 epidemic is also an important factor leading to the deterioration of the mental health of adolescents [4]. Mental health problems in children and adolescents continue to affect mental health in adulthood, and school sports in adolescence can prevent mental health problems in adulthood [5]. Research has shown that COVID-19 is associated with lower levels of physical activity, lower self-esteem, and higher social anxiety. Whether the relationship between the change of physical activity level and self-esteem, social anxiety has changed after the COVID-19 epidemic deserves further exploration [6]. Therefore, this paper explores the internal relationship between physical activity and mental health of adolescents after the COVID-19 epidemic, and whether social anxiety and self-esteem mediate these relationships.

Numerous studies have found that participation in physical activity has a positive impact on adolescent mental health, and that physical activity predicts mental health in college students [7]. However, in a follow-up survey of 4023 subjects, Vella found that there was also a two-way relationship between participation in sports and adolescent mental health [8]. Guddal’s research on the relationship between physical activity and mental health in adolescents of different ages found that high levels of physical activity were positively associated with various aspects of mental health, particularly for high school adolescents [9]. The outbreak of COVID-19 epidemic has a significant impact on mental health, especially young people are more vulnerable to the impact during the COVID-19 epidemic, causing mental health problems [10]. Although there have been some studies on the relationship between youth participation in physical activity and mental health, the study of the relationship between youth participation in physical activity and mental health after the epidemic also has certain significance.

Self-esteem refers to an individual’s attitude toward self-worth, an attitude that leads the individual to believe that he or she is worthy and capable [11]. Research shows that self-esteem is significantly associated with mental health and predicts health status in adolescents [12], Male self-esteem is significantly higher than female self-esteem [13]. In studies of narcissism and self-esteem related to comprehensive adolescent mental health, high and high-moderate self-esteem characteristics were found to be associated with adolescent mental health, while low self-esteem was associated with psychological problems [14]. The relationship between self-esteem and mental health has been well documented in numerous studies. There is a significant positive relationship between physical activity and self-esteem [15]. David’s analysis of female pre university sports participation, enjoyment, and self-esteem found that female students’ pre university sports participation can moderately predict self-esteem levels, and female participants with unpleasant sports experiences may be at risk of decreased self-esteem [16]. A study shows that women have less physical activity and lower self-esteem than men during the COVID-19 epidemic. The association between self-esteem and mental health and the relationship between self-esteem and physical activity have been demonstrated through various studies, and a few studies have explored the mediating role that self-esteem plays in physical activity and mental health. For example, Bang et al. found that extracurricular activities played an important role in reducing depressive symptoms by increasing school engagement and self-esteem [17]. Therefore, according to previous studies, it can be predicted that after the COVID-19 epidemic, the increase in physical activities that adolescents participate in can further promote mental health by improving adolescents’ self-esteem.

Social anxiety refers to the experience of pain, uneasiness, fear, anxiety, etc., in social situations; deliberate avoidance of social situations; fear of negative evaluation of oneself by others [18]. It has been shown that social anxiety is a cause of mental health problems and negatively affects mental health through two different mechanisms:reduced satisfaction and reduced psychological flexibility [19]. Physical activity can improve the level of social anxiety in adolescents. Kliziene found that physical education courses have a good effect on physical anxiety, personality anxiety and social anxiety by studying the impact of physical education courses on the physical activity and emotional health of pupils for four months [20]. A study have shown that the isolation state during the COVID-19 epidemic reduced people’s social interaction, leading to a significant increase in people’s social anxiety, while changes in physical activity did not affect social anxiety [6]. There are differences in the impact of changes in physical activity on social anxiety between the epidemic period and the non epidemic period, so whether changes in physical activity after the COVID-19 epidemic can affect social anxiety, and whether social anxiety plays a mediating role between youth sports activities and mental health is worth further exploring.

**Fig 1:**
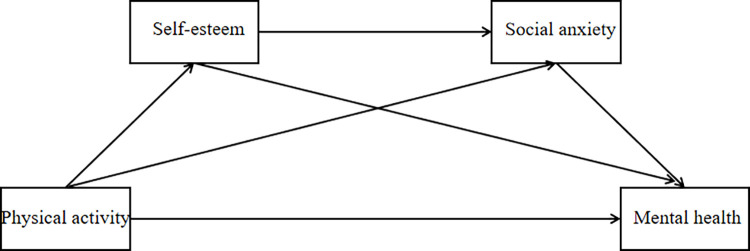
Hypothetical model diagram.

In previous studies, social anxiety was found to be negatively associated with self-esteem [21], Self-esteem can reduce social anxiety [22], and self-esteem mediated the relationship between social anxiety and other variables. Ciarma found that self-esteem mediates the association between social anxiety and eating disorders in his study [23], Rasmussen found that self-esteem partially mediated the association between positive thinking and social anxiety in his study of the direct and indirect benefits of positive thinking about personality on self-esteem and social anxiety [24], and Ran found that self-esteem partially mediated the association between shyness and social anxiety in his study [25]. According to the basis of previous studies, it can be hypothesized that physical activity affects adolescent mental health first through self-esteem and then through social anxiety (Fig 1).

## 2. Method

All the experiments were performed in accordance with the Declaration of Helsinki Ethical Principles for Medical Research Involving Human Subjects and other relevant laws, regulations, and ethical norms. This study was approved by the Ethics Committee of Shandong University (approval number: 61351342/2020–389).

### 2.1. Participants

This study was conducted from July to September 2023, a cross-sectional study was conducted using convenience sampling to collect data from 400 middle-school students in Jinan, Shandong province. We have obtained the consent of the participants, and all parents are required to fill out an electronic version of the informed consent form before the survey. After agreeing to participate, students will receive an electronic questionnaire that they need to complete independently.

After excluding invalid questionnaires, 359 valid questionnaires were obtained, and the effective recovery rate was 89.75%. The survey population was between 12 and 16 years old (M = 13.74, SD = 1.067), with 200 males (55.7%) and 159 females (44.3%), with 257 (71.6%) newly crowned infections and 102 (28.4%) uninfected newly crowned infections, 141 (39.3%) students were in the first year, and 99 (27.6%), and 119 (33.1%) students were in the third year.

### 2.2. Measures

#### 2.2.1. Physical activity

The measurement of physical activity adopts a dimension in the HPLP - Ⅱ. Health Promotion Lifestyle Scale. The HPLP - Ⅱ is a revised version of the HPLP, which is composed of 52 items, and still has a six factor structure:sports, health responsibility, stress management, nutrition, interpersonal relations and spiritual growth. This study adopts the sports dimension, with each item assigned a score of 1, 2, 3, and 4 from "never", "sometimes", "often", and "always", with a score of 1–4. The higher the score, the higher the level of physical activity of the participants. In this study, the Cronbach’s alpha of the questionnaire was 0.881.

#### 2.2.2. Self-esteem

The self-esteem scale was developed by Rosenberg, and includes three parts: behavioral self-esteem, social self-esteem, and instrument self-esteem. The scale consists of 10 questions and is divided into 5 rating levels. The higher the score, the higher the self-esteem level. The lower the score, the lower the self-esteem level, with a maximum score of 40 points. If the measurement result is greater than 25 points, the participate is considered to have high self-esteem. The scale has high reliability and validity, and has been verified to be suitable for measuring the self-esteem of Chinese college students. In this study, the Cronbach’s alpha of the questionnaire is 0.638.

#### 2.2.3. Social anxiety

Social anxiety was measured using the SPIN Social Phobia Inventory, which investigated the subject’s social anxiety-related problems for the past week, including three dimensions of fear, avoidance, and physical discomfort, with 17 self-rated questions, each rated on a scale from 0 to 4, with scores ranging from 0 to 68, and with a score of 19 as the threshold value for the presence or absence of social anxiety, which has been shown by previous studies to be good In the present study, the Cronbach’s alpha of the questionnaire was 0.958.

#### 2.2.4. Mental health

The mental health survey was conducted using the Kessler Psychological Distress Scale, which consists of 10 items and focuses on evaluating the frequency of anxiety, depression, fear, and other negative emotions in the past 1 month after the individual has been stimulated by stress, sadness, etc. Using a Likert scale, 5 = "all the time", 4 = "most of the time", 3 = "some of the time", 2 = "occasionally ", and 1 = "almost never". The score range was 10 to 50, with higher scores indicating higher psychological distress. Scores of 30 to 50 indicated severe psychological distress, 22 to 29 indicated moderate psychological distress, and 10 to 15 indicated almost no psychological distress. The Cronbach’s alpha was 0.964 in our sample, suggesting good internal consistency.

### 2.3. Data analysis

SPSS.27 was used for descriptive statistics, correlation analysis, questionnaire reliability testing, and chain mediation model analysis. After processing each study variable, model 6 in the macro program PROCESS plug-in in SPSS was used to test the chain mediating effect of self-esteem and social anxiety between physical activity and mental health and the chain mediating effect of self-esteem and social anxiety respectively.

## 3. Results

### 3.1. Common method deviation test

The Harman single-factor test method was used to conduct factor analysis on all items involved in this study. The results showed that exploratory factor analysis extracted a total of 18 factors with feature values greater than 1, and the variation explained by the first factor was 29.25%, far below the critical value of 40%. Common method bias did not affect the data in this study.

### 3.2. Descriptive analysis and correlation analysis

The results of descriptive statistics as well as correlation analysis of the study variables are shown in Table 1. Descriptive statistics found that 359 secondary school students had physical activity scores between 8 and 32 (M = 20.53, SD = 5.39), mental health scores between 10 and 50 (M = 40.61, SD = 8.74), self-esteem scores between 0 and 40 (M = 28.51, SD = 3.57), and social anxiety scores between 0 and 68 (M = 30.39, SD = 13.38). Association analysis showed that physical activity was positively associated with mental health (r = 0.223, p<0.01), physical activity was significantly positively associated with self-esteem (r = 0.338, p<0.01), and physical activity was negatively associated with social anxiety (r = -0.298, p<0.01). Mental health was positively associated with self-esteem (r = 0.561, P<0.01), mental health was negatively associated with social anxiety (r = -0.583, P<0.01), and social anxiety was negatively associated with self-esteem (r = -0.537, P<0.01). Age was negatively associated with physical activity (r = -0.207, P<0.01), grade was negatively associated with physical activity (r = -0.211, P<0.01), and mental health (r = -0.124, P<0.05), and gender was negatively associated with physical activity (r = -0.157, P<0.01), and positively associated with social anxiety (r = 0.148, P<0.01).

**Table 1:** Results of descriptive statistics and correlation analysis.

	M	SD	1	2	3	4	5	6	7
Physical activity	20.53	5.39	1.000						
Mental health	40.61	8.74	0.223**	1.000					
self-esteem	28.51	3.57	0.338**	0.561**	1.000				
Social anxiety	30.39	13.38	-0.298**	-0.583**	-0.537**	1.000			
Age	13.74	1.033	-0.207**	-0.066	-0.085	0.047	1.000		
Grade	/	/	-0.211**	-0.124*	-0.095	0.095	0.866**	1.000	
Gender	/	/	-0.157**	-0.072	-0.079	0.148**	-0.010	0.038	1.000

*P<0.05

**P<0.01

### 3.3. Analysis of differences between groups

With COVID-19 infection as the grouping variable, this study explored whether COVID-19 infection had an impact on the mental health of adolescents. The results are shown in Table 2 that there was no significant difference in mental health, self-esteem or social anxiety between the adolescents infected with COVID-19 and those not infected with COVID-19. The difference between the mental health level of adolescents infected with COVID-19 and those not infected with COVID-19 was the largest, but not significant. The results showed that whether adolescents were infected with COVID-19 did not affect their mental health, self-esteem or social anxiety. This result proves that hypothesis H5 is not valid.

**Table 2:** Analysis of differences between groups with and without COVID-19 infection.

Variables	Infected with COVID-19	Not infected with COVID-19	t	p
Physical activity	20.46±5.298	20.71±5.64	-0.38	0.497
Mental health	40.86±8.663	39.96±8.943	0.871	0.727
self-esteem	28.65±3.659	28.15±3.322	1.265	0.339
Social anxiety	30.23±13.201	30.8±13.891	-0.361	0.279

*P<0.05

**P<0.01

### 3.4. Analysis of mediating effects

**Fig 2:**
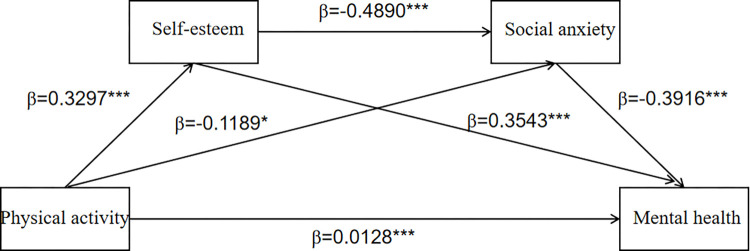
Chain intermediary model. ***p<0.001.

Using SPSSPROCESS, a bias-corrected percentile bootstrap was used to estimate 95% confidence intervals for each effect, controlling for gender, age, and grade, followed by a test for mediating effects. The mediation effect results are shown in Table 3 that the indirect effect of the path with self-esteem as the mediating variable was 0.1894 (95% CI = [0.1079, 0.2779]), the indirect effect of the path with social anxiety as the mediating variable was 0.0755 (95% CI = [0.0033, 0.1702]), the indirect effect of the path with self-esteem and social anxiety as the mediating variable was 0.1023 (95% CI = [0.0539, 0.1706]), and the total indirect effect of all indirect effects was 0.3672 (95% CI = [0.2401, 0.5075]). Based on the above results and Fig 2, we found that self-esteem plays a more significant mediating role in adolescent physical activity and mental health than social anxiety does, and that the chain mediating role of self-esteem and social anxiety in the effect of adolescent physical activity on mental health holds. The total effect of physical activity on adolescent mental health was significant (β = 0.3335, p<0.01), and the relationship between physical activity and adolescent mental health weakened after adding self-esteem and social anxiety as mediating variables and was no longer significant (β = -0.0337, p>0.05), therefore, self-esteem and social anxiety played a fully mediating role in the effect of physical activity on adolescent mental health (Fig 2).

**Table 3:** Results of the test for mediating effects of youth physical activity on mental health.

Effect	Path Correlation	Effect Value	Confidence interval
Direct effect	PA→MH	-0.337	[-0.1750, 0.1076]
Intermediary Effect	PA→SE→MH	0.1894	[0.1079, 0.2779]
	PA→SA→MH	0.0755	[0.0033, 0.1702]
	PA→SE→SA→MH	0.1023	[0.0539, 0.1706]
Total inter mediation effect		0.3672	[0.2401, 0.5075]
Total effect		0.3335	[0.1635, 0.5034]

## 4. Discussion

The purpose of this study is to verify and analyze the internal relationship between physical activity and self-esteem, social anxiety and mental health of adolescents after the COVID-19 epidemic. The study found that self-esteem and social anxiety play a mediating role in the association between physical activity and adolescent mental health, and that self-esteem and social anxiety play a chain mediating role in the association between physical activity and adolescent mental health.

The results of the study showed a positive predictive effect of physical activity on mental health in the adolescent population, i.e., active participation in physical activity is beneficial to adolescent mental health development, verifying hypothesis 1. This result is consistent with previous studies that have concluded that physical activity can improve adolescent mental health [7, 26]. A literature review on physical activity and mental health in children and adolescents showed a small but consistent association between physical activity and mental health [27]. In addition, participation in physical activity has an antidepressant effect [28]. This means that physical activity is a preventive factor for mental health and can increase positive psychological outcomes for participants, such as self-efficacy, and resilience [29]. The results of the study showed that the higher the grade level of schooling, the lower the participation in physical activity and the lower the level of mental health of the youth. According to the endorphin hypothesis, participation in physical activity promotes the release of endorphins in the body, which makes participants happy and thus serves to prevent psychological problems such as depression [30], and adolescents in lower grades participate in more physical activity and release higher levels of endorphins, thus exhibiting higher levels of mental health.

Self-esteem and social anxiety play a fully mediating role between physical activity and mental health, i.e., physical activity influences mental health exclusively through self-esteem and social anxiety. The same previous research that self-esteem mediates the association between physical activity and adolescents’ psychological well-being tested Hypothesis 2. Specifically, sports participation was a significant predictor of self-esteem [17]. Adolescents who participate in sports show higher levels of self-esteem than those who do not participate in sports [31], Participation in sports improves adolescents’ self-esteem levels through increased athleticism, improved physical abilities, and improved appearance self-concepts [32]. After the COVID-19 epidemic, adolescents’ participation in physical activity increased significantly, and their level of self recognition of the body improved relatively, thus further improving their self-esteem [33]. Stress theory mentions that higher levels of self-esteem can reduce stress by mitigating perceived threats and choosing effective coping strategies to improve mental health [34]. Additionally, research has shown that cheerleading activities can significantly improve the physical self-esteem level of female college students and further improve their overall mental health [35]. This study emphasized the importance of participating in physical activity to improve adolescents’ self-esteem after the COVID-19 epidemic, and the mediating role of self-esteem between sports activities and mental health.

Social anxiety played a small but significant mediating role between physical activity and adolescent mental health, a result that validated Hypothesis 3. The research report said that the more adolescents participated in physical activity after the COVID-19 epidemic, the lower the level of social anxiety. This is consistent with previous research results showing that a school physical education curriculum can effectively improve students’ social anxiety [36]. Multiple experimental studies have shown that team sports, and recreational activities can reduce social anxiety levels in adolescents [37, 38]. The study also reported that teenagers’ mental health was threatened by social anxiety, and the increase of physical activity after the COVID-19 epidemic reduced adolescents’ social anxiety. Similar to previous studies, social anxiety is a negative influence on adolescent mental health [19], and women show more social anxiety symptoms [39]. Inconsistent with previous research the study did not find an association between age and social anxiety. Therefore, social anxiety is a risk factor for adolescent mental health, and physical activity is a protective factor for adolescent social anxiety and mental health.

The results of the study indicate the chain mediating role of self-esteem and social anxiety in the effects of physical activity on adolescent mental health. This result tested Hypothesis 4 that physical activity influences adolescent mental health through the chain-mediated role of self-esteem and social anxiety. Self-esteem mediates the relationship between physical activity and social anxiety in the chain-mediated pathway between physical activity and mental health. The results of the study reported a significant negative association between self-esteem and social anxiety, a result that is consistent with the cognitive behavior theory of social anxiety, which suggests that low self-esteem is a major cause of social anxiety [40]. People with high self-esteem have high self-evaluations and are more active in interpersonal interactions, whereas people with low self-esteem have low self-evaluations and are more passive in interpersonal interactions [41]. Thus, adolescents with low self-esteem have high levels of social anxiety, and adolescents with higher levels of self-esteem have low levels of social anxiety [42], that is higher self-esteem may act as a protective factor against social anxiety [43]. Physical activity increases participants’ self-esteem levels by increasing their sense of self-worth [16], and increased self-esteem levels positively affect participants’ social anxiety. Lower levels of social anxiety make adolescents more proactive in their interpersonal interactions, which promotes healthy associations between adolescents and their peers, and healthy associations with peers contribute to adolescent mental health, thus helping adolescents to develop psychologically health [44].

This study has certain limitations. First, the cross-sectional study design limited the ability to confirm causal relationships between physical activity, self-esteem, social anxiety and mental health. Therefore, in-depth investigations in conjunction with experimental intervention studies and longitudinal studies are needed to further explore the mechanisms of action between the variables. Second, this study relied on self-reports and despite passing the common method bias test, there was some social and subjective bias. Although the reliability of the questionnaire was good, a combination of subjective and objective methods could be used in future studies to avoid the problem of biased measurements. Third, the sample for this study was drawn from one region of China, thus limiting the generalisability of the findings. Despite the limitations of the study, this study expands the study on the endogenous mechanisms of the impact of physical activity on mental health, elucidating multiple mediating pathways for the impact of physical activity on mental health.

## 5. Conclusion

The purpose of this study was to explore the influence mechanism of physical activity on adolescents’ mental health through self-esteem and social anxiety after the COVID-19 epidemic. The findings suggest that self-esteem and social anxiety can serve not only as separate mediators of the associations between physical activity and mental health, but also as chain mediators of the associations between physical activity and mental health. These findings have important implications for the development of adolescent mental health, with self-esteem as one of the core variables protecting the development of adolescent mental health.

Most studies show that the decrease of physical activity during the COVID-19 period may lead to mental health problems of adolescents [2]. Some studies also show that there is a no significant relationship between physical activity changes and other psychological indicators during the COVID-19 period [6]. This study explored the changes in the relationship between physical activity and mental health of the two groups based on whether they were infected with COVID-19. Due to the long-term nature and uncertainty of the COVID-19 epidemic, its impact on the physical activity and mental health of young people needs to be further explored.
